# A multi-point decentralized control for mitigating vibration of flexible space structures using reaction wheel actuators

**DOI:** 10.1038/s41598-024-60702-6

**Published:** 2024-05-06

**Authors:** Chen Bifa, Jianbin Liao, Jin Yan, Hanlin Li, Chaoming Huang

**Affiliations:** 1 School of Marine Engineering, Jimei University, Xiamen, China; 2 Fujian Institute of Innovation for Marine Equipment Detection and Remanufacturing Industrial Technology, Xiamen, China

**Keywords:** Flexible space structure, Reaction wheel actuator, Multi-point actuation, Decentralized control, Aerospace engineering, Civil engineering, Mechanical engineering

## Abstract

The vibration with large amplitude and low frequency of the flexible space structures is prone to affect the attitude stability and pointing precision of the spacecraft. To mitigate the vibration of the flexible space structures, a multi-point decentralized control strategy using reaction wheel (RW) actuators is proposed and investigated in this paper. The motion equations of the solar array with multiple RW actuators are derived in modal coordinate representation. To suppress the overall response of the structure, the decentralized control strategy using RW actuators is designed based on the natural frequencies and mode shapes. The stability and the effect of closed-loop dynamic system is theoretically proved. The comparative studies under sun-pointing of the solar array and the rest-to-rest orbital maneuver conditions are presented to show the control performance of the RW actuators. The results indicate that, with 2% increase in total mass from the addition of the actuators, the vibration attenuation time can be decreased by 85.25% and 94.16% for the vibration excited by the sun-pointing and the rest-to-rest orbital maneuver, respectively. The experimental results demonstrate the effectiveness of the proposed decentralized control method. Theoretical analysis, numerical simulation and experimental study are conducted to demonstrate the validity of the proposed vibration mitigation approach and its potential application in the spacecraft design.

## Introduction

To meet the increasing demands of deep space exploration and applications, the space flexible appendages/structures, such as solar array, truss antenna, and robotic manipulators are tending to be light-weighted and have large deployed scale and low stiffness^1,2^. The low-frequency vibrations the flexible structures are likely to be excited by the various on-orbit operations. Due to the lack of the air and the weak friction under the low gravity environment in space, it takes a long time for the vibrating structures to be sufficiently attenuated. The long-term vibrations not only influence the pointing stability and precision of the spacecraft but also lead to the structural damage accumulation^3,4^. Therefore, it is urgently needed to consider the vibration suppression problems of the flexible space structures.

For decades, many scholars have proposed various methods for the vibration issue of the flexible space structures. The methods can primarily be divided into the passive control and the active control^5–7^. The passive control is applied by adding damping elements or modifying the physical properties of the structure to reduce vibration^7,8^. Owing to the advantages of cost-effectiveness and non-interference with the system stability, the passive control was firstly applied to the vibration suppression of spacecraft flexible appendages. A classical case was the installation of passive dampers between the drive mechanism and the root of the solar panels on the Hubble Space Telescope^9^. However, for the low-frequency vibration of the flexible structures, the natural frequencies are extremely low, and the additional damping of the passive control can be limited. The effect of passive control on reducing vibration attenuation time is not significant. Balas and Shepherd^10^ had concluded that the active control was necessary for the vibration suppression of spacecraft flexible structures.

The active control system is formed by combining the sensors, controllers, and actuators. The process involves acquiring the dynamic signals by sensors, solving the control signals by the designed algorithms, and supplying the appropriate magnitude and direction of actuating forces to the vibrating structure by actuators. Selecting the suitable actuators for different application situation is a key issue in active vibration control. The piezoelectric materials are a kind of the most frequently-used actuators in active control^11–14^. For example, Tarazaga et al.^15^ achieved a good vibration reduction effect by using the MFC as sensors and actuators in the space truss structures. But for the large-scale flexible appendages, the vibration suppression effect of the piezoelectric materials is not obvious due to their limited actuating capability. This is because the allowed adhesive area of piezoelectric materials is very small compared to the large-sized flexible appendages, which leads to limiting the actuation effect. Sun et al.^16^ proposed an active vibration control scheme for solar panels by using the cable-driven actuators. The control law was established by making a trade-off between the actuating force and the vibration suppression effects. It should be noted that, for the deployable space structures, the involved cables require reconsideration of their deployment process design and its robustness. D’Eleuterio et al.^17^ used the control moment gyroscope (CMG) as the actuator. The CMG actuators were mounted on the vibrating structure to form the so-called gyroelastic body. The actuating force, i.e., the gyroscopic torque, was induced by changing the orientation of the CMG and applied to the structure for vibration suppression. The optimization of the control strategies and the dynamic modeling methods were continuously proposed and improved for vibration suppression of the flexible space structures^18–20^. However, such methods need to control the rotation speed of CMG and its orientation simultaneously, which requires a relatively complex control system in real application. Moreover, the mounted CMG actuator induce a significant impact on the modal vibration of the controlled structure^21,22^. Wu et al.^23^ utilized a reaction wheel to suppress vibration of the solar array by the reaction torque induced by the wheel speed change. This control method has the advantage of simplifying the actuator structure, system dynamic modeling, as well as the control procedure. Meanwhile, they achieved a good vibration reduction result.

The trend of larger size, lower stiffness, and more complex configuration in spacecraft flexible appendages has led to a focus on how to suppress the overall system response^24,25^. While the aforementioned literatures mainly focused on the lateral bending vibration in single direction of flexible space structures. Few focused on the strategies to suppress vibrations of the whole system with multi-point actuation. The distributed control provides an effective solution for suppressing the systematic vibration with multi-point actuation. The key aspect in the distributed control design is to build the information interaction between various control submodules. Zhou et al.^26^ conducted studies on application of the distributed control in the vibration suppression of solar panels. Researchers have considered the scalability of control and adopted a mode of adjacent submodules exchanging measurement information with each other. Omidi et al.^27^ developed an Integral Consistency Controller (ICC) for the vibration control of flexible structures embedded with piezoelectric stacks. This controller consists of $N$ independent controllers corresponding to $N$ control submodules on the structure. By connecting these control submodules under a specific directed communication graph, the ICC eliminates disagreements between controllers and enhances their fault tolerance. Tian et al.^28^ proposed a actuator location optimization criteria, and adopted a distributed cooperative control mode with multiple actuators. The effectiveness of the method was verified through simulations. Due to the requirement of information interaction between different submodules in distributed control, it not only increases computational costs and affects control efficiency in practice, but also puts higher requirements on control robustness and stability. Once a submodule failure occurs, it is likely to disrupt the overall collaborative action of distributed control. The decentralized control can have better robustness and achieve overall system vibration suppression through independent feedback control with multiple actuators.

The previous paper focused on the lateral bending mode vibration of solar array, and the single actuator used was suitable for this type of problem. This paper aims to seek a multi-actuator control method to solve the complex modal vibration modes and their overall response vibration. Therefore, this article promotes and upgrades the aforementioned method, introducing complex modal vibration control based on the decentralized multi-point actuation, and applying the method to the overall bending and torsional modal vibration response of more complex multi-panel deployable solar array. The reaction torque, induced by the wheel speed change, is controlled to damp the vibration of the solar array for multi-point actuation with decentralized control. For the sake of implementation, the control of the reaction torque of RW actuator is transformed to the control of its rotating speed through integral. A multi-panel deployable solar array with RW actuators is investigated to validate the effectiveness of the proposed method.

The rest of the paper is organized as follows. In "Mechanical principles of vibration suppression with multi-point actuation" section, The mechanical principles of the solar array actuated by multiple RW actuators are elucidated theoretically. In "Multi-point decentralized control strategy for RW actuators" section, the decentralized controller of the RW actuators is designed, and the stability of the closed-loop dynamic system is demonstrated based on the Lyapunov function. A real-scale multi-panel deployable solar array is taken as the object to verify and evaluate the effectiveness of the proposed method through numerical simulations in "Numerical simulation and verification" section. Experimental results are presented in "Experimental research" section to illustrate the effectiveness and feasibility of the presented method. Conclusions are drawn in "Conclusion" section.

## Mechanical principles of vibration suppression with multi-point actuation

**Figure 1:**
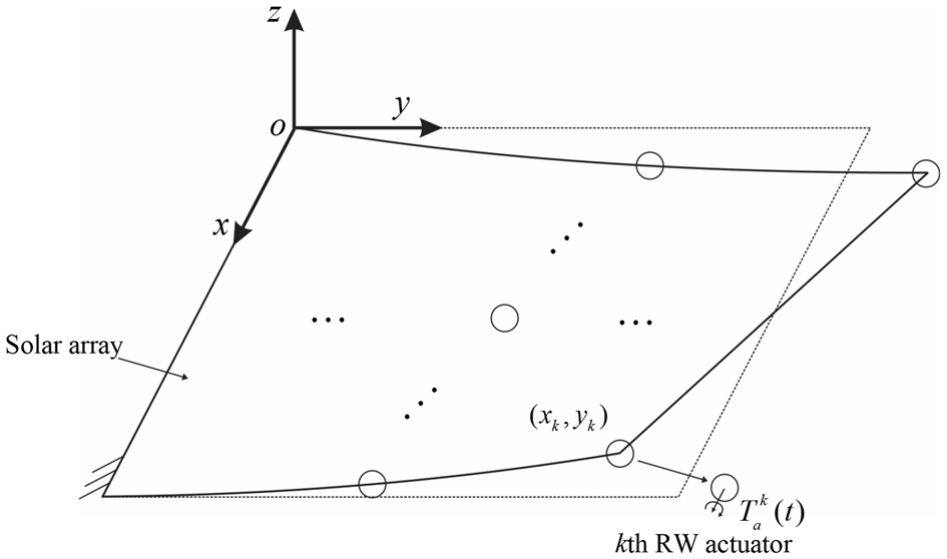
Structural diagram of the solar array with RW actuators.

Taking a solar array with the RW actuator as the object, the solar array is corresponding to an elastic cantilever plate with the clamped root, as shown in Fig. 1. Denote the mounted position of the $k$th RW stator as $(x_{k} ,y_{k} )$. According to the theorem of moment of momentum, the reaction torque is induced by changing the rotor speed. Thus. the reaction torque applied to the solar array by the $k$th RW can be written as:

1 $$T_{a}^{k} (t) = - I\dot{\Omega }^{k} ,$$

where $I$ is the moment of inertia of the wheel rotor. $\Omega^{k} ,\dot{\Omega }^{k}$ are the rotational speed and corresponding angular acceleration of the rotor, respectively.

Once disturbed, the vibration of the cantilever plate is dominated by the mode shape laterally perpendicular to the $xy$ surface. The number of the RW actuators mounted on the solar array is $K$. The direction of the wheel rotation axis is parallel to the $x$-axis. The Dirac $\delta -$function is used to express the actuating placement of the RW actuators. According to the theory of transverse vibration of plates^29^, the deflection function $w(x,y,t)$ of the cantilever plate satisfies the partial differential equation:

2 $$\begin{aligned} & D_{s} \nabla^{2} \nabla^{2} w + \rho h\ddot{w} \\ & \quad = \sum\limits_{k = 1}^{K} {\delta (y - y_{k} )\delta^{\prime}(x - x_{k} )T_{a}^{k} (t)} , \\ \end{aligned}$$

where $\nabla^{2} = \frac{{\partial^{2} }}{{\partial x^{2} }} + \frac{{\partial^{2} }}{{\partial y^{2} }}$ is the Laplace operator; $D_{s} = \frac{{Eh^{3} }}{{12(1 - \upsilon^{2} )}}$ is the bending stiffness of the plate; $h,\rho ,E,\upsilon$ are the thickness, mass density, elastic modulus, and Poisson's ratio of the plate, respectively. $\delta (x)$ is the Dirac $\delta -$function, and a single prime point ^′^ represents the first derivative with respect to $x$.

By separating variables, the deflection function can be expressed as a function of the modal coordinate $\xi_{mn}$:

3 $$w(x,y,t) = W_{mn} (x,y)\xi_{mn} (t),$$

in which, $W_{mn} (x,y)$ is the spatial function subjected to the boundary conditions. Especially, for the cantilever plate shown in Fig. 1, $W_{mn} (x,y)$ can be written as the product of the free beam mode function $X_{m} (x)$ along the x-axis and the cantilever beam mode function $Y_{n} (y)$ along the *y*-axis^30^, i.e., $W_{mn} (x,y) = X_{m} (x)Y_{n} (y)$. For convenience of illustration, the two-dimensional subscript is represented by a one-dimensional subscript, that is, the $mn$th order mode of the overall vibration of the cantilever plate is denoted as the $i$th order mode, and the spatial function using a one-dimensional subscript is denoted as $\varphi_{i} (x,y) = W_{mn} (x,y)$. Therefore, Eq. (3) can be rewritten into:

4 $$w(x,y,t) = \sum\limits_{i = 1}^{\infty } {\varphi_{i} (x,y)\xi_{i} } = {{\varvec{\Phi}}}^{{\text{T}}} {{\varvec{\upxi}}}.$$

Substitute Eq. (4) into Eq. (2), and considering the characteristic equation $D\nabla^{2} \nabla^{2} {{\varvec{\Phi}}} = \rho h{\mathbf{\Omega \Phi }}, \, {{\varvec{\Omega}}} = {\text{diag}}\{\omega_{i}^{2}\}$, in which, $\omega_{i}$ is the $i$th natural frequency. One can obtain:

5 $$\begin{aligned} & \rho h{{\varvec{\Phi}}}^{{\text{T}}} ({\mathbf{\ddot{\xi }}} + {\mathbf{\Omega \xi }}) \\ & \quad = \sum\limits_{k = 1}^{K} {\delta (y - y_{k} )\delta^{\prime}(x - x_{k} )T_{a}^{k} (t)} . \\ \end{aligned}$$

Multiplying ${{\varvec{\Phi}}}$ on both sides of Eq. (5) and integrating along the surface of the plate lead to:

6 $${\mathbf{\ddot{\xi }}} + {\mathbf{\Omega \xi }} = {\mathbf{M}}^{ - 1} {\mathbf{f}}(t),$$

where

7 $${\mathbf{M}} = \iint {\rho h{\mathbf{\Phi \Phi }}^{{\text{T}}} {\text{d}}x{\text{d}}y} \triangleq {\text{diag}}\{M_{i}\} ,$$

8 $$\begin{aligned} {\mathbf{f}}(t) = & \iint {{{\varvec{\Phi}}}\sum\limits_{k = 1}^{K} {\delta (y - y_{k} )\delta^{\prime}(x - x_{k} )T_{a}^{k} (t)} {\text{d}}x{\text{d}}y} \\ = & \sum\limits_{k = 1}^{K} {T_{a}^{k} (t)\frac{{\partial {{\varvec{\Phi}}}(x_{k} ,y_{k} )}}{\partial x}} . \\ \end{aligned}$$

Introduce the damping matrix expressed by ${{\varvec{\Lambda}}} = {\text{diag2}}\{\omega_{i} \zeta_{i}\}$. Preserve the first $n_{K}$ order modal coordinates of Eq. (6). The differential equation of motion represented by the $i$th modal coordinate $\xi_{i}$ can be expressed as:

9 $$\ddot{\xi }_{i} + 2\zeta_{i} \omega_{i} \dot{\xi }_{i} + \omega_{i}^{2} \xi_{i} = \sum\limits_{k = 1}^{K} {\varphi_{{{\theta ,}i}}^{k} T_{a}^{k} } ,$$

where

10 $$\varphi_{{{\theta ,}i}}^{k} = \frac{{\partial \varphi_{i} (x_{k} ,y_{k} )}}{\partial x}/M_{i} .$$

According to Eq. (9), if the modal parameter $\varphi_{{{\theta ,}i}}^{k}$ is not equal to zero, it is theoretically possible to suppress the $i$th order principle vibration by designing the reaction torque $T_{a}^{k}$ through the control law. Furthermore, Eq. (9) also indicates that, to obtain the modal force $\varphi_{{{\theta ,}i}}^{k} T_{a}^{k}$ as much as possible and maximize the vibration suppression effect, it is critical to place the RW actuator in a region of large displacement. As derived above, the present vibration control method is applied to the flexible structures conform to the assumption of isotropy.

## Multi-point decentralized control strategy for RW actuators

For the large flexible structures, the vibrations are basically dominated by the low-order modes^31^. Therefore, in this paper, the undesired vibration mode to be suppressed is designated as the $i$th mode of the overall vibration of the solar array. Based on the motion differential Eq. (9), the control law of the RW actuators is designed for vibration suppression.

### Controller design and stability analysis

It is preferred to consider as many modes as possible to control vibration when designing the controller. However, this will require more controller resources and reduce the computational efficiency. The independent modal control method is adopted, and the $i$th mode is utilized for controller design without loss of generality. To achieve the vibration reduction effect, the generalized force $\varphi_{{{\theta ,}i}}^{k} T_{a}^{k}$ on the right side of Eq. (9) can be designed to act as the artificial damping. Therefore, the control law for the reaction torque of the RW actuator can be designed to be proportional to the time derivative of modal coordinate $\dot{\xi }_{i}$, which is written as:

11 $$\varphi_{{{\theta ,}i}}^{k} T_{a}^{k} = - \chi_{k} \dot{\xi }_{i} \;\;(\chi_{k} > 0),$$

where $\chi_{k}$ is a positive proportion coefficient, the negative symbol makes $\varphi_{{{\theta ,}i}}^{k} T_{a}^{k}$ in the opposite direction of $\dot{\xi }_{i}$. For a better suppression effect, the control gain $\chi_{k}$ should be increased as much as possible on the premise that the actuators are not overloaded. The reaction torque of $k$th RW actuator is thus given by:

12 $$T_{a}^{k} = - \frac{{\chi_{k} }}{{\varphi_{{{\theta ,}i}}^{k} }}\dot{\xi }_{i}$$

The Eq. (12) is the control law implemented in this paper.

Overall, the control law (12) is employed for the RW actuators to suppress vibration of the solar array. When $T_{a}^{k} = 0$, the open-loop system is asymptotically stable, as it is lightly damped. The proposed feedback control law given in Eq. (11) is to increase the structural damping effect, hence the closed-loop system stability can be guaranteed. It is worth noting that, although the controller is designed using reduced-order models, it is applied, in the numerical simulation, to full-order mode based on finite element method for verification. Meanwhile, to minimize the impact on higher-order modes, a bandpass filter is utilized to filter out the residual modal signal components. For the linear structures, the modal coordinate $\xi_{i}$ can be solved by measuring physical quantities, such as displacement, bending moment, or strain, etc., as the input of the control system.

### Transfer function model of RW actuators

Let $M_{{\text{b}}}$ be the measured bending moment of the solar array by sensors. In numerical simulation, the torsion spring elements are used to model the torque sensors to measure the bending moment. Thus, the relative angular displacement at both ends of the element is proportional to the bending moment. The bending moment $M_{{\text{b}}}$ can be expressed by the local rotational angle $\theta_{r}$ in the same direction, or approximately by the modal coordinate as $M_{{\text{b}}} = k_{{\text{b}}} \theta_{{\text{r}}} \approx \varphi_{{\text{M}}} \xi_{i}$. Here, $\varphi_{{\text{M}}}$ is a constant related to the modal function. Combining Eqs. (1) and (12), the angular acceleration of the wheel can be obtained as follows:

13 $$\dot{\Omega }^{k} = \eta \dot{M}_{{\text{b}}} ,$$

in which

14 $$\eta = \frac{{\chi_{k} }}{{I\varphi_{{\text{M}}} \varphi_{{{\theta ,}i}}^{k} }}.$$

It is favorable for engineering application to achieve angular speed control of the RW actuators rather than angular acceleration control. Thus, integrating Eq. (13) on both sides about time $t$ leads to:

15 $$\Omega^{k} = \eta M_{{\text{b}}} .$$

It can be seen from Eq. (15) that the control law, expressed in Eq. (12), can be transformed into the control of the wheel speed $\Omega^{k}$ to induce needed torque.

In real application, the measured bending moment should be filtered to decrease the undesired high-frequency and the zero-drift noise. Thus, the digital signal denoising process with a specific filter is considered. In this paper, a second-order Butterworth bandpass filter with a passband of $\omega_{i} \times [0.6,\;1.4]$ is adopted. As given in Eq. (18), a proportional factor is used to establish the control relationship between the bending moment $M_{{\text{b}}}$ and the wheel rotation speed $\Omega$.

As mentioned, the measured signal is filtered to obtain the component containing unexpected modal natural frequencies. However, it leads to the phase delay of the filtered signal. When using the modal truncation method to design the controller, the phase delay may poses a risk of high-order modal divergence in the control process. Therefore, the derivative link is involved for phase compensation of the filtered signal. For linear time-invariant structures, the natural frequency of the unexpected mode is constant, and thus, the phase angle does not change over time. The derivative gain $D$ is given by:

16 $$D = - \frac{\tan \gamma }{{\tilde{\omega }}}.$$

Here $\gamma$ is the phase angle of filter; $\tilde{\omega }$ is the natural frequency of the vibration mode to be suppressed.

The controller for vibration suppression is therefore a PD (proportional-derivative) controller. The transfer function between the reaction torque $T_{a}^{k}$ and the measured bending moment $M_{b}$ in Laplace domain can be expressed as:

17 $$\mathcal{L}[T_{a}^{k} ] = \eta Is(1 + sD)G_{{\text{c}}} (s)\mathcal{L}[M_{{\text{b}}} ],$$

where, $\mathcal{L}[ \cdot ]$ represents the Laplace transform; $s$ is the Laplace variable; $G_{{\text{c}}} (s)$ is the transfer function of the filter. Equation (17) can be supplemented to the finite element model of the solar array to establish the closed-loop control system.

## Numerical simulation and verification

In this section, the proposed method is verified by using a multi-panel deployable solar array as the application object. The modal analysis of the solar array is firstly performed to acquire the natural modes and their corresponding frequencies. The numerical simulation of the vibration response of the solar array under the on-orbit loads are conducted by finite element method. The vibration characteristics of the solar array are identified from the frequency response. The aforementioned controller design method is implemented for RW actuators to achieve vibration suppression. Finally, the effectiveness of the proposed method is evaluated and verified.

### Finite element model and vibration characteristics analysis

**Figure 2:**
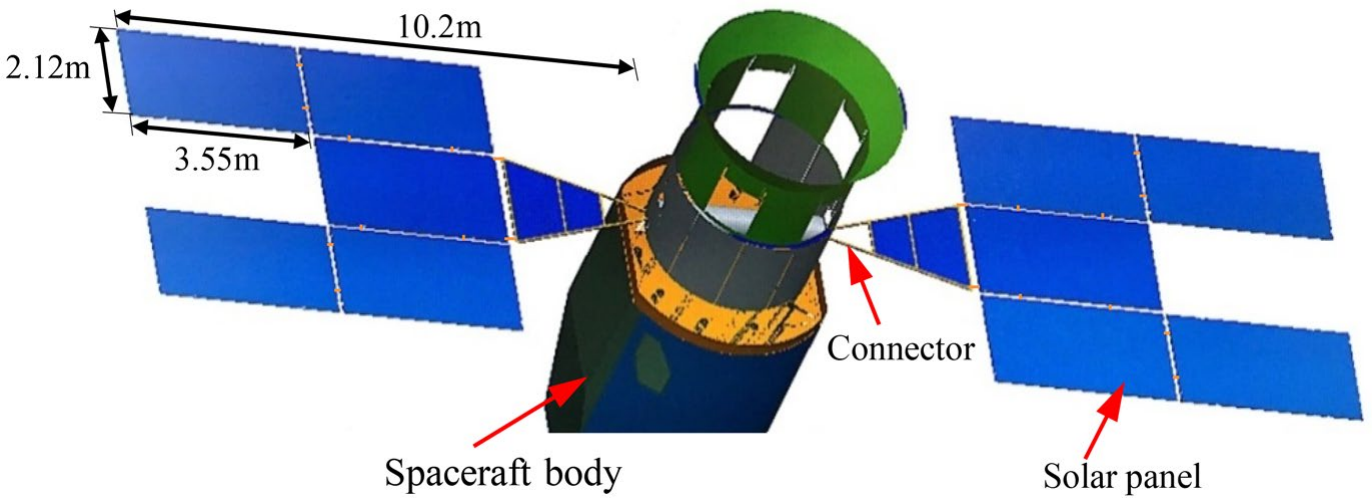
The spacecraft with multi-panel deployable solar array.

The multi-panel deployable solar array consists of 5 rectangular panels and 1 trapezoidal panel is taken as the object, as shown in Fig. 2. The span dimension along the deploying direction is 10.2 m, and the area of the single rectangular solar panel is $3.55{\text{ m}} \times {2}{\text{.12 m}}$. A Cartesian coordinate system $O - XYZ$ is established, with the origin $O$ located at the root where the solar array connects to the spacecraft. The *Z*-axis is aligned with the deploying direction, the *Y*-axis is perpendicular to the panel surface, and the *X*-axis follows the right-hand rule.

**Figure 3:**
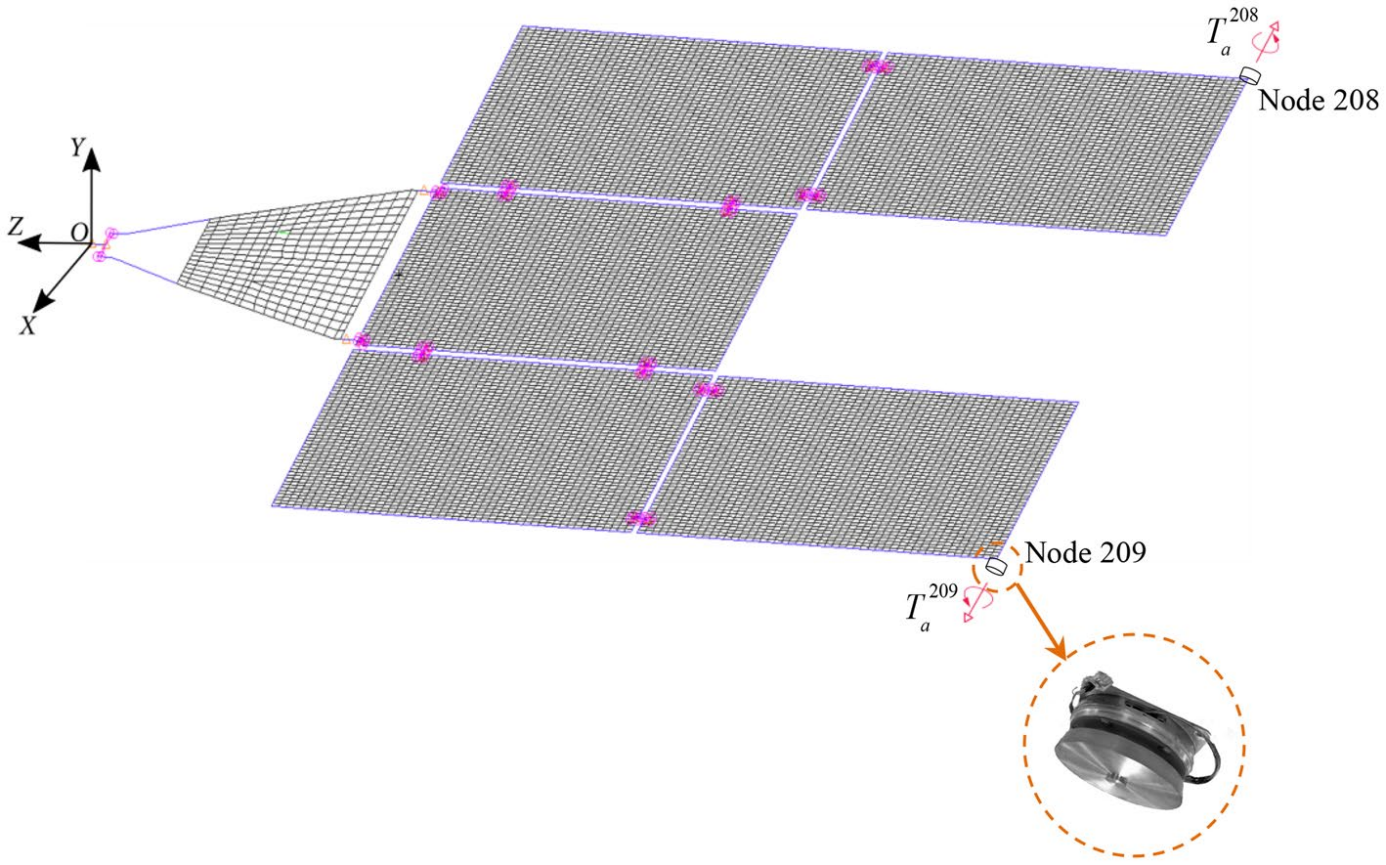
The diagram of the finite element model of the multi-plate deployable solar array with RW actuators.

The corresponding finite element model of the solar array is constructed for numerical study and it is shown in Fig. 3. The solar panels are modeled using shell elements. The panel-to-panel connected structures and the supported framework are modeled with beam elements. The total mass of the solar array, including non-structural mass, is 181.61 kg. At the connection between the root of the solar array and the spacecraft body, the torsion spring elements with a modulus $k_{{\text{b}}} { = }10^{6} \;{\text{ N}}\;{\text{m/rad}}$ are used to simulate moment sensors around the *X*-, *Y*-, and *Z*-axis, respectively. The other directions are assumed to be rigidly connected.

**Figure 4:**
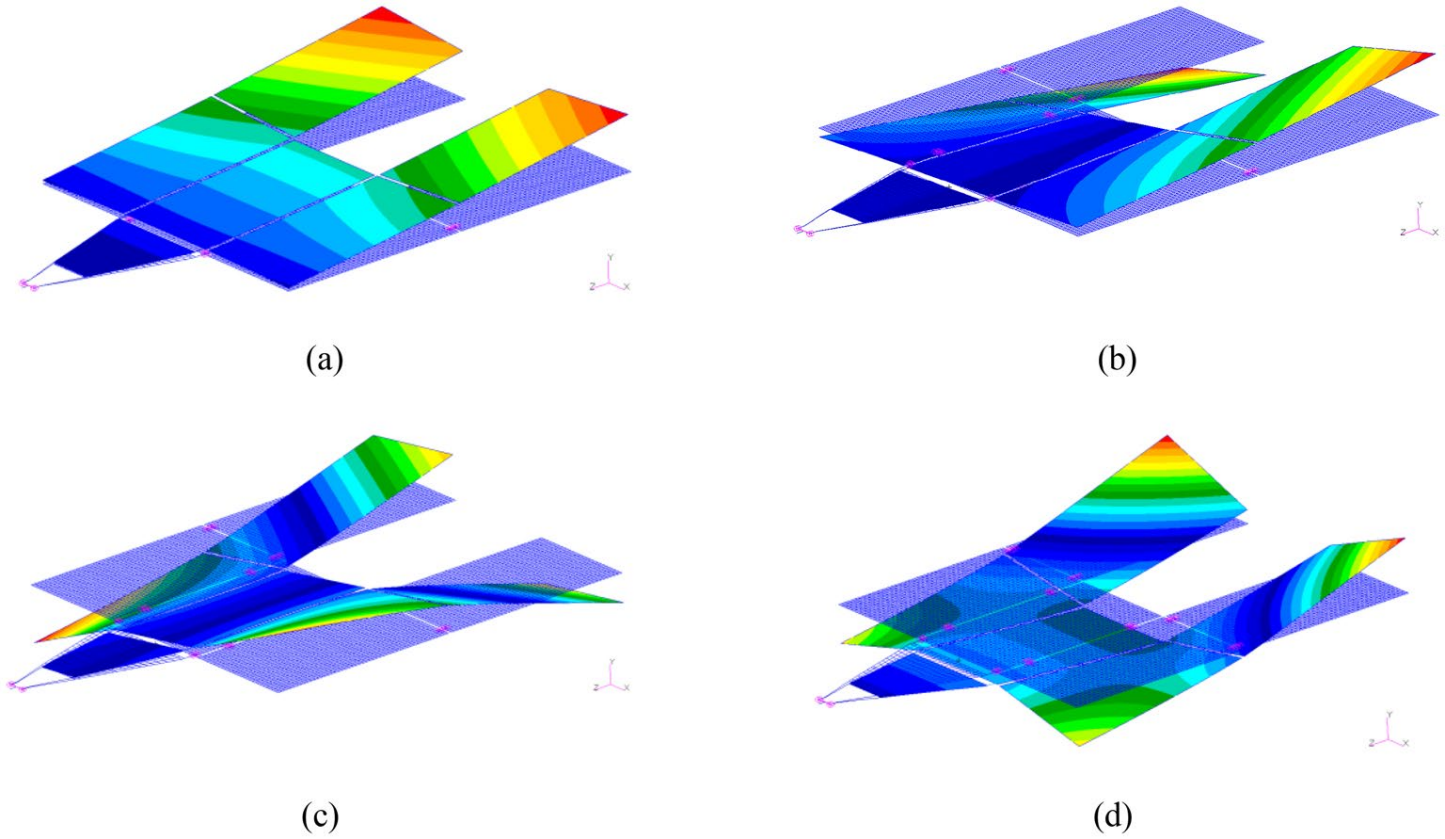
Natural frequencies and the corresponding modes of solar array (**a**) First bending (0.153 Hz), (**b**) first torsional (0.179 Hz), (**c**) second torsional (0.519 Hz) and (**d**) second bending (0.542 Hz).

Modal analysis is performed for the solar array to obtain the first four natural frequencies and their corresponding mode shapes, as shown in Fig. 4. The transient response of the finite element model of the solar array is analyzed through numerical simulations. Thus, a Rayleigh damping model is employed, and expressed as:

18 $${{\varvec{\Lambda}}} = \alpha {\mathbf{I}} + \beta {{\varvec{\Omega}}}.$$

The coefficients $\alpha ,\beta$ can be acquired according to:

19 $$\left\{ \begin{gathered} \alpha + \beta \omega_{m}^{2} = 2\omega_{m} \zeta_{m} \hfill \\ \alpha + \beta \omega_{n}^{2} = 2\omega_{n} \zeta_{n} \hfill \\ \end{gathered} \right., \, m \ne n.$$

Based on the testing experience of the similar structures in engineering, let $m = 1,n = 2$ and the damping ratios of the first two modes of the solar array are taken as $\zeta_{1,2} = 0.005$, respectively. The damping parameters are solved by Eq. (19), and one can obtain $\alpha { = 4}{\text{.6}} \times 10^{ - 3} ,\;\;\beta { = 5}{\text{.4}} \times 10^{ - 3}$.

The mass of the RW actuator is limited within 2 kg, which accounts for approximately 1% of the total mass of the solar array. Referring to the relevant motor product, the concrete parameters of RW actuators mentioned in simulation are listed as follows, the moment of inertia of the rotor is $I = 0.00{5 }\;{\text{kg}}\;{\text{m}}^{2}$, and the maximum rotational speed is $\Omega^{\max } { = 261}{\text{.7994 rad/s}}$. As illustrated in Eq. (9), to provide the modal forces $\varphi_{{{\theta ,}i}}^{k} T_{a}^{k}$ as much as possible, the stators of the RW actuators are mounted at two corner nodes, i.e., Node 208 and Node 209, respectively. The bearing axis of the RW actuators are parallel to the *X*-axis, as shown in Fig. 3.

### Vibration suppression of solar array under on-orbit loads

#### Case 1: Sun-pointing of solar array condition

The solar array is in full-deployed configuration when on orbit. To maximize the effective insolation area and make the normal direction of the panel surface aligned with the direction of incident sunlight, the solar array can rotate around the *Z*-axis through the driving mechanism at its root. The sun-pointing orientation is a highly-frequently used on-orbit operation.

**Figure 5:**
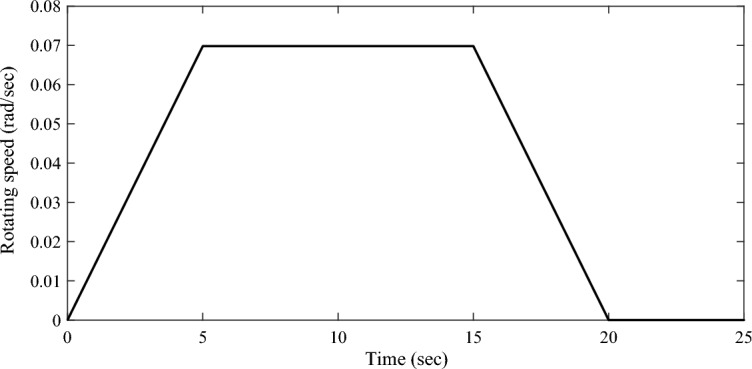
The rotating speed of the driving mechanism in sun-pointing.

In this simulation, the driving mechanism is administrated by the "uniform acceleration-uniform velocity-uniform deceleration" trapezoidal function, as shown in Fig. 5. The rotating speed of the driving mechanism around the *Z*-axis is given by:

20 $$Rotating \,speed = \left\{ {\begin{array}{*{20}c} {{0}{\text{.01396}}t,\,\,\,\,\,\,\,\,{ 0} \le t < 5} \\ {0.0698,\,\,\,\,\,\,\,{ 5} \le t < 15} \\ {0.0698 - {0}{\text{.01396}}t,\,\,\,\,\,\,\,{ 15} \le t < 20} \\ {0, \, t < 0{\text{ or }}t \ge 20} \\ \end{array} } \right.$$

It should be noted that, Eq. (20) guarantees the whole sun-pointing process lasts for 20 s, and the solar array rotates $60^{ \circ }$ around the *Z*-axis.

**Figure 6:**
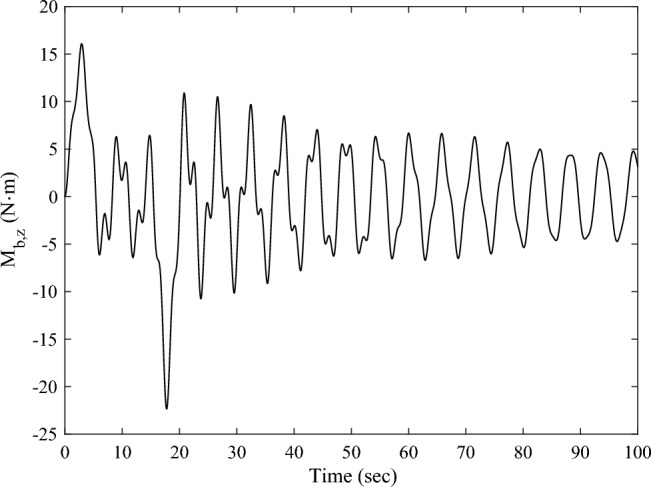
Time-domain response of the root bending moment of the solar array in sun-pointing.

**Figure 7:**
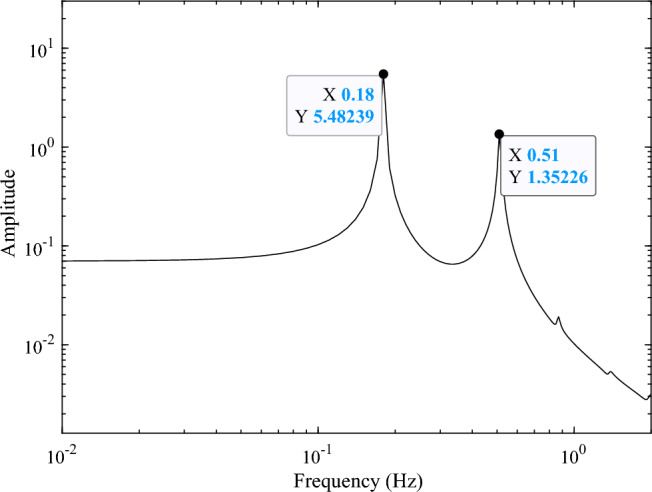
Frequency-domain response of the root bending moment of the solar array in sun-pointing.

The residual vibration response analysis of the solar array excited by the sun-pointing process is shown as Fig. 6. The corresponding frequency response is shown as Fig. 7. Figure 7 illustrates that, the excited vibration is dominated by the first-order torsional mode (see in Fig. 4b).

Referring to Eq. (11), the reaction torques of the RW actuators can be designed in equal magnitude and opposite direction, which is written as:

21 $$T_{a}^{208} = - T_{a}^{209} = - \frac{{\chi_{208} }}{{\varphi_{{{208},{\uptheta }}} }}\dot{\xi }_{2} .$$

Here, $T_{a}^{208}$ and $T_{a}^{209}$ represent the reaction torques induced by the RW actuators mounted at Node 208 and Node 209, respectively. It should be noted that, the equal magnitude and opposite direction of $T_{a}^{208}$ and $T_{a}^{209}$ are determined by its modal characteristics, and the control law can be designed independently.

Considering the signal-to-noise ratio of the sensing signals and the results of modal analysis, the measured bending moment is arranged at the connection position between the root of the solar array and the spacecraft body around the *Z*-axis. The measured bending moment is designated as $M_{{{\text{b}},z}}$, and utilized as the input of the control system (17).

**Figure 8:**
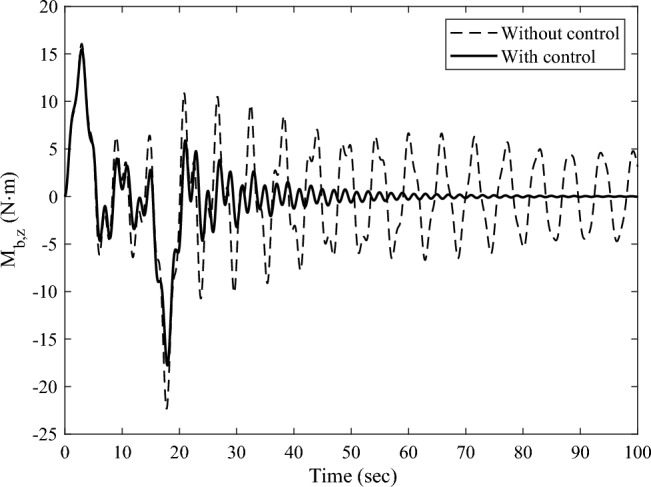
The root bending moment response with and without control in sun-pointing.

**Figure 9:**
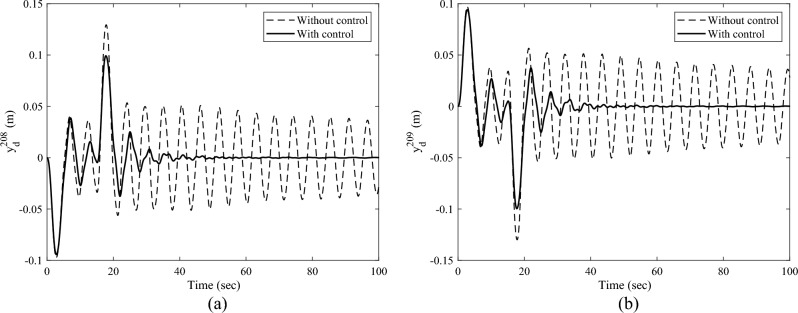
The translational displacement responses along *Y*-axis at reaction wheel mounted node with and without control in sun pointing (**a**) displacement response of Node 208 and (**b**) displacement response of Node 209.

**Figure 10:**
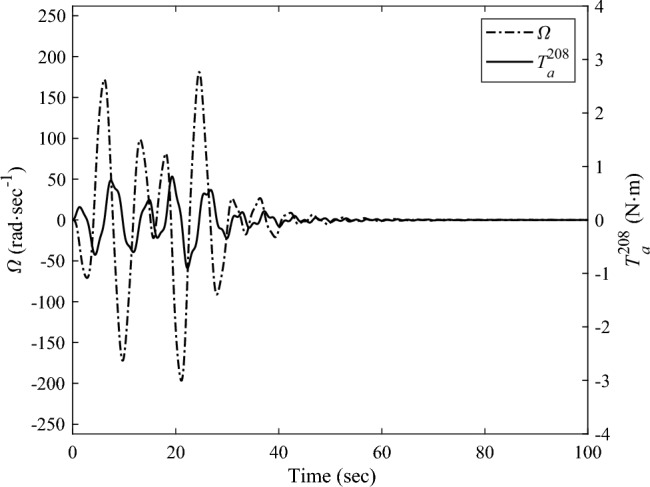
The rotating speed and reaction torque output by RW actuator mounted at Node 208 in sun-pointing.

Denote the displacements of the Node 208 and Node 209 along the $Y -$ axis as $y_{{\text{d}}}^{208}$,$y_{{\text{d}}}^{209}$, respectively. Here, the root bending moment $M_{{{\text{b}},z}}$ of the solar array, as well as $y_{{\text{d}}}^{208}$ and $y_{{\text{d}}}^{209}$ are focused and plotted to analyses the response of the solar array. After completing the transient response calculation and data processing, the variation curves of $M_{{{\text{b}},z}}$, $y_{{\text{d}}}^{208}$,$y_{{\text{d}}}^{209}$ over time are obtained, as shown in Figs. 8 and 9a, b, respectively. The black dashed and the black solid lines represent the vibration attenuation curves without control and with control, respectively. Taking the root bending moment as an example, let the threshold value be $M_{{{\text{b}},z}}^{*} { = }\,{2}\;{\text{N}}\;{\text{m}}$. The time required for the root bending moment $M_{{{\text{b}},z}}$ of the solar array to attenuate to the threshold $M_{{{\text{b}},z}}^{*}$ is 244.8 s without control, and 36.1 s with control. Compared to the state without control, the vibration attenuation time with control is reduced by 85.25%, which demonstrates the effectiveness of the multi-point actuation method through RW actuators. The variation curves of the wheel speed $\Omega$ and the reaction torque $T_{a}^{208}$ in the control process over time are shown as the dashed and solid lines in Fig. 10, respectively. The peak value of the wheel speed is $196.9\;{\text{ rad/s}}$, and the maximum reaction torque is $0.9 \, \;{\text{ N}}\;{\text{m}}$, both of which are within the rated range.

#### Case 2: Rest-to-rest orbit maneuver condition

**Figure 11:**
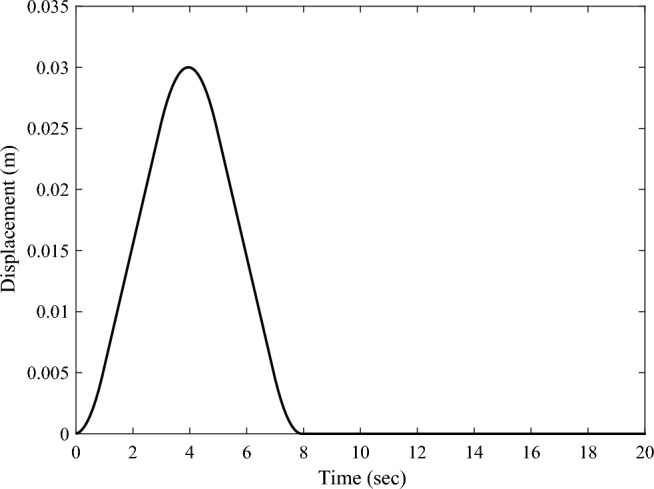
The root translational displacement function in orbital maneuver.

**Figure 12:**
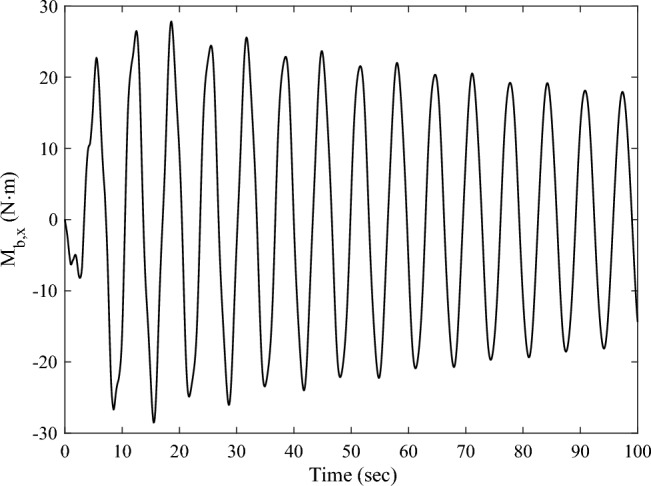
Time-domain response of the root bending moment of the solar array in orbital maneuver.

**Figure 13:**
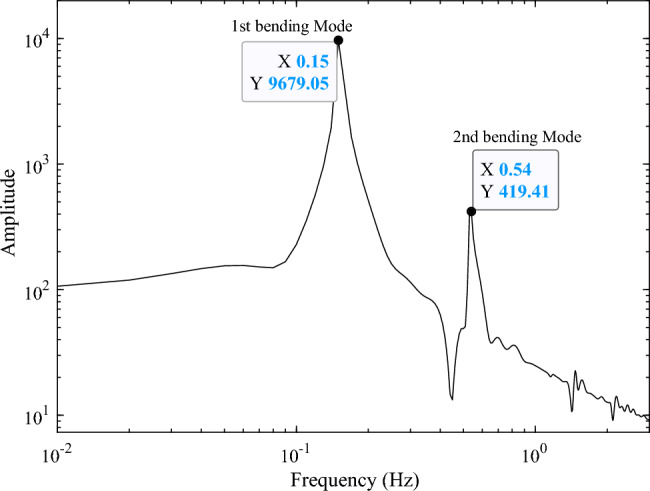
Frequency-domain response of the root bending moment of the solar array in orbital maneuver.

The forced displacement at the root of the solar array along the *Y*-axis is adopted to simulate the rest-to-rest orbit maneuver condition by using a triangular function. The forced root displacement function over time is shown in Fig. 11 with a period of 8 s and an amplitude of 0.015 m. The transient response of the solar array under the excitation is calculated, and the root bending moment response curve around the *X*-axis $M_{b,x}$ is obtained. The time-domain response data in Fig. 12 is employed for frequency spectrum analysis, and the corresponding frequency-domain response curve of the bending moment $M_{b,x}$ is shown in Fig. 13. The analysis reveals that under the orbit maneuver condition, the vibration of the solar array is dominated by the bending mode, especially the first bending vibration. The mode shape is shown in Fig. 4a.

According to modal symmetry $\varphi_{{{208},{\uptheta }}} { = }\varphi_{{{209},{\uptheta }}} \ne 0$, the reaction torques are designed to be of equal magnitude and in the same direction:

22 $$T_{a}^{208} = T_{a}^{209} = \frac{{\chi_{208} }}{{\varphi_{{{208},{\uptheta }}} }}\dot{\xi }_{1} .$$

Here, the input of the control system (17) is the root bending moment $M_{{{\text{b}},x}}$ around the *X*-axis. The measured point of $M_{{{\text{b}},x}}$ is arranged at the connection position between the root of the solar array and the spacecraft body.

**Figure 14:**
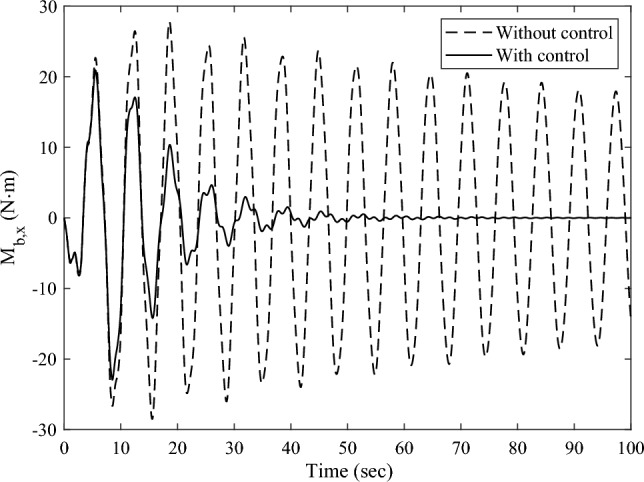
The root bending moment response with and without control in orbital maneuver.

**Figure 15:**
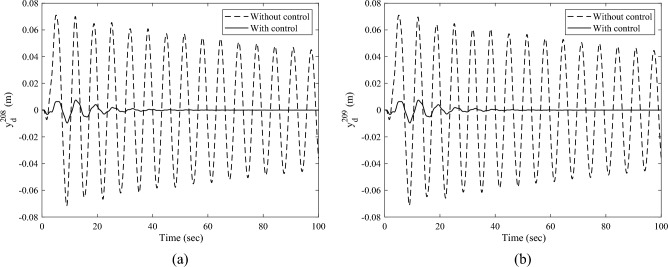
The translational displacement responses along Y-axis at reaction wheel mounted node with and without control in orbital maneuver (**a**) displacement response of Node 208 and (**b**) displacement response of Node 209.

**Figure 16:**
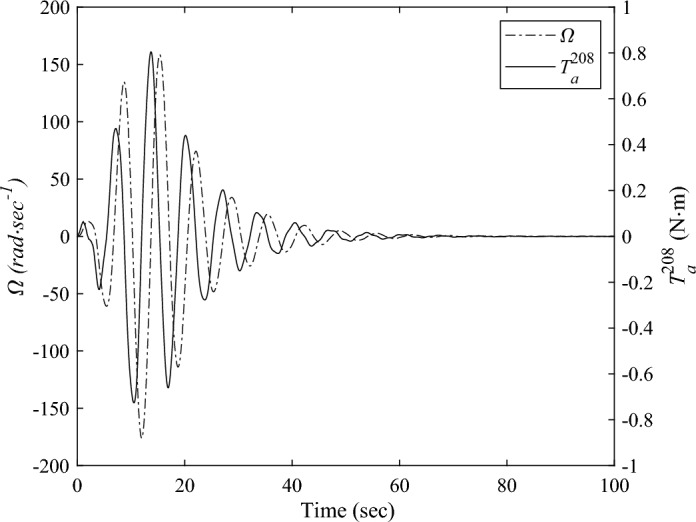
The rotating speed and reaction torque of the reaction wheel mounted at Node 208 in orbital maneuver.

The transient response analysis is conducted after applying control and data processing. The variation curves $M_{{{\text{b}},x}}$,$y_{{\text{d}}}^{208}$,$y_{{\text{d}}}^{209}$ respect to time are obtained and shown as Figs. 14 and 15a, b respectively. The black dashed and solid line represent the vibration attenuation curve without and with control, respectively. It takes 556.3 s for the root bending moment $M_{{{\text{b}},x}}$ to decay to the threshold value $2{\text{ N}} \cdot {\text{m}}$ without control. While it takes 32.5 s after applying control. The vibration attenuation time is reduced by 94.16%, which verifies the effectiveness of the multi-point actuation method for vibration suppression The variation curves of the wheel speed $\Omega$ and reaction torque $T_{a}^{208}$ with respect to time during the control process are shown by the dashed line and solid line in Fig. 16, respectively. Both the wheel speed and reaction torque remain within the rated range.

## Experimental research

### Experimental setup

**Figure 17:**
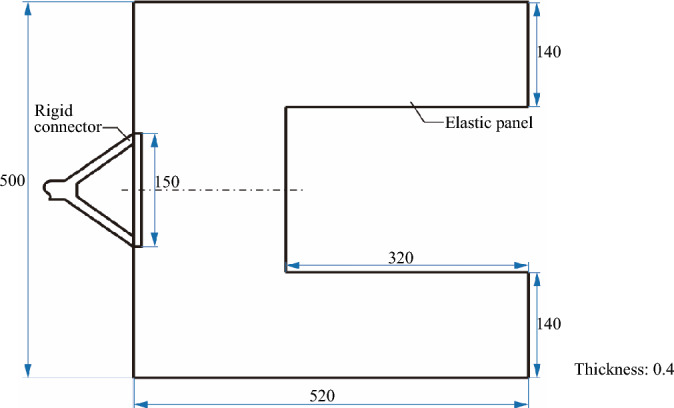
Dimensions of the elastic panel (units: mm).

**Figure 18:**
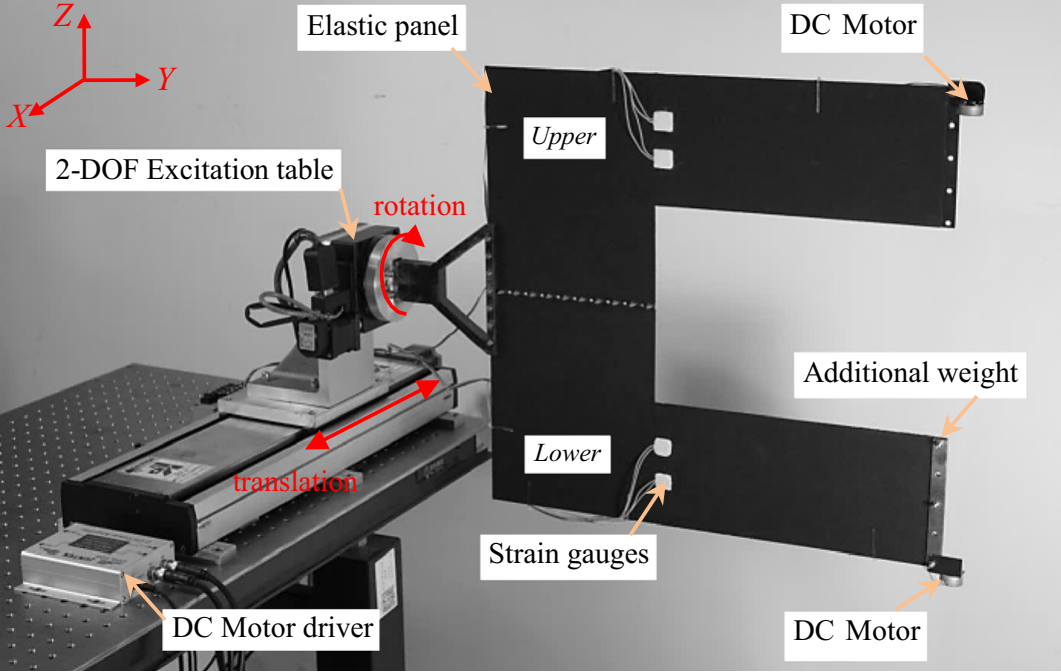
Experimental setup for vibration mitigation.

In this section, the experiment study is carried out for verification of the vibration mitigation measure. Due to the gravity and air on the ground have a significant impact on large deployable flexible structures, a small-sized and simplified experimental model is used in the laboratory to verify the control method of vibration suppression of solar array. The U-shaped elastic panel made of spring steels is used as the test object. The basic dimensions of the panel are shown in Fig. 17. Figure 18 shows the components of the experimental setup.

As shown in Fig. 18, the elastic panel is laterally placed to reduce the influence of gravity. One side of the panel is fixed on a 2-DOF excitation table through a rigid connector. The excitation table excites the U-shaped elastic panel from the translation along the *X*-axis and the rotation around the *Y*-axis, respectively. The other side is mounted with two brush-DC motors (BDCMs), which serve as RW actuators to supply reaction torques. In this case, the total mass of the DC motor is 17 g, and its rotor and stator mass are 5.4 g and 11.6 g, respectively. Namely, the mass ratio of the rotor to stator is 0.465. Additional weight and anti-shock sponge with a mass of 97.4 g and a thickness of 0.5 mm are installed on the panel to adjust the vibration frequency and damping of the experiment model, respectively. The first two order frequencies of the experimental model are 0.89 Hz and 1.03 Hz, corresponding to the bending and torsional vibration modes, respectively.

In the experiment, the strain gauges with a resistance of $350{\Omega }$ are bonded on the elastic panel to acquire the local bending moment signal. As shown in Fig. 18, there are the upper and the lower channels of bending moment signal to capture the local dynamic responses. The input voltage of the full-bridge strain circuit is 10 V. The output voltage is amplified 1000 times linearly by strain amplifier and sampled by the A/D (Analog-to-Digital) module. Let strain voltages $V_{{\upvarepsilon }}^{u} ,V_{{\upvarepsilon }}^{l}$ be the strain amplifier’s output voltage changes of the upper and the lower channels, respectively. Through the A/D module, the corresponding digital values of the strain voltages are input to the control system.

When the elastic panel is disturbed, $V_{{\upvarepsilon }}^{u}$ and $V_{{\upvarepsilon }}^{l}$ change with the vibration of the elastic panel. According to the pre-designed control law algorithm, the signal voltages, which command the speed of the DC motors, are solved and output to the motor driver through a D/A (Digital-to-Analog) module. There are two channels of signal voltage, as well, i.e., the upper and the lower channels, which are denoted as $V_{{{\text{dri}}}}^{u}$ and $V_{{{\text{dri}}}}^{l}$. In this research, the speed signal voltage range of the motor driver is $\pm 6{\text{ V}}$ corresponding to the speed $\pm 4000{\text{ RPM}}$ of DC motor.

To simulate the disturbance from the spacecraft body, the excitation is exerted as follows. The excitation directions are along the direction perpendicular to the panel surface and around the extension direction, respectively. The speed functions of the sliding and rotating table are given by:

23 $$\left\{ \begin{gathered} v_{t} = 0.01\sin (2\pi \times 0.9t)\;{\text{ m/s}} \\ v_{r} = 2\pi /180\sin (2\pi \times 0.9t)\;{\text{ rad/s}} \\ \end{gathered} \right.,$$

respectively. The excitation period is 2/0.9 s.

The bending moment signals are measured by strain gauges during the entire time period, i.e., from the beginning of the excitation to the end of the experiment. In order to verify the effectiveness of the active control strategy, the vibration attenuation with and without control are both measured. It should be noted that, for the linear structures, to reflect the structural vibration attenuation process with and without control in the experiment, respectively, the measured the dynamic response, i.e., the strain voltages $V_{{\upvarepsilon }}^{u} ,V_{{\upvarepsilon }}^{l}$, are plotted.

### Experimental result

**Figure 19:**
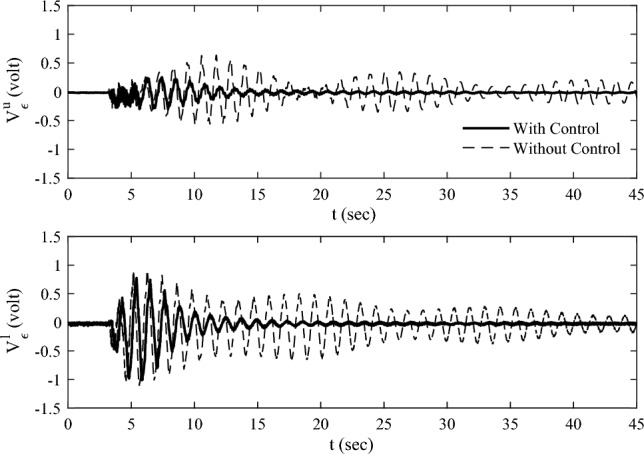
The measured strain voltages of the elastic plate.

**Figure 20:**
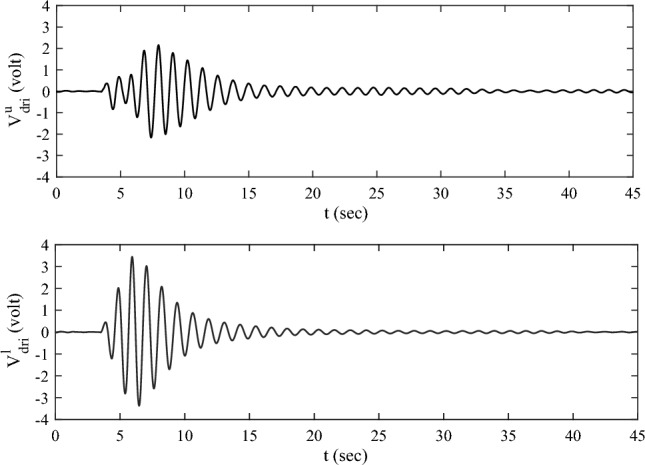
The speed signal voltages of the motors.

The measured strain voltage of the elastic plate $V_{{\upvarepsilon }}^{u}$ and $V_{{\upvarepsilon }}^{l}$ are shown in Fig. 19. The solid line is corresponding to the response with control. The dashed line is corresponding to the response without control. As can be seen, the vibration attenuation with control is faster than the one without control. This result illuminates the validity of the multi-point actuating control scheme by using RW actuators. The measured speed signals $V_{{{\text{dri}}}}^{u} ,V_{{{\text{dri}}}}^{l}$ are given in Fig. 20, which reveals that the actuators can operate in the rated operating range during control. $V_{{{\text{dri}}}}^{u} ,V_{{{\text{dri}}}}^{l}$ converge to zero with the attenuation of vibration.

## Conclusion

In this paper, a multi-point decentralized control method using multiple RW actuators is proposed for mitigating the overall vibration of the flexible space structures with complex mode shape. Both the simulation results and the experimental data are provided to demonstrate that the effect of the multi-point decentralized control is significant for suppressing vibration of the flexible space structures. The actuating forces induced by the wheel speed change are designed and applied to the structures to suppress the undesired vibration. Based on the plate theory and Dirac delta function, the motion equations of the dynamic system represented in modal coordinates are derived for controller design. By controlling the actuating forces to be proportional to the time derivative of the modal coordinates, the proposed decentralized control for increasing the structural artificial damping is proved to be stable theoretically. The criterion of the control parameter calculation is determined based on the modal characteristics of the flexible space structures. Especially, it is found from the simulation results that, for a real-scale multi-panel deployable solar array under on-orbit load, the vibration attenuation time is decreased by 85.25% and 94.16% for sun-pointing and orbit maneuver conditions, respectively. The proposed methodology provides the new possibilities to the vibration, where an effective and synchronized suppression is needed.

## Data Availability

Data will be made available on request from the corresponding author (Bifa Chen: 202361000021@jmu.edu.cn).
